# Fluid signal changes around the knee on MRI are associated with increased volumes of subcutaneous fat: a case-control study

**DOI:** 10.1186/s12891-016-1345-8

**Published:** 2016-11-24

**Authors:** Gaunt Trevor, Frank Carey, John Cahir, Andoni Toms

**Affiliations:** Norwich Radiology Academy, Norfolk and Norwich University Hospitals NHS Foundation Trust, Colney Lane, Norwich, Norfolk NR4 7UB UK

**Keywords:** Subcutaneous fat, Subcutaneous fluid, Obesity, Fluid signal, Knee, Pain, Shear forces, Shearing, T2 hyper-intensity

## Abstract

**Background:**

Fluid in the subcutaneous fat is a common finding anterior to the knee on MRI. This may be caused by chronic low-grade shearing injuries in patients who are overweight. The purpose of this study was to determine if there is a difference in the amount of subcutaneous fat around the knee between patients with these appearances and controls.

**Methods:**

This was a retrospective case-control study. Following a sample size calculation on pilot data, eighteen sequential patients demonstrating hyper-intense subcutaneous signal changes around the knee on fat-saturated T2-weighted MRI were identified from PACS (18 females, mean age 45, range 31–62). Age and gender-matched patients without abnormal T2 MR signal changes were selected. Two observers independently drew regions of interest representing cross-sectional areas of bone and fat. The location of T2 signal hyper-intense lesions was characterized by consensus.

**Results:**

Inter and intra-rater intraclass reproducibility was “excellent” (ICC > 0.8). The mean cross-sectional area of bone for patients with T2 hyper-intense lesions was 31.79cm^2^ (SD 2.57) and for controls 30.11cm^2^ (SD 3.20) which was not significantly different (*p* = 0.09). The median cross-sectional area of fat for the study group was 62.29cm^2^ (IQR 57.1–66.5) and for controls was 32.77cm^2^ (IQR 24.8–32.3) which was significantly different (*p* < 0.0001). Consensus agreement demonstrated all T2 hyper-intense lesions were anterior to the knee extensor mechanism.

**Conclusion:**

Subcutaneous fluid around the knee is associated with an increased amount of subcutaneous fat, anterior to the knee extensor mechanism. This may be caused by shearing injuries in fat with reduced elasticity associated with metabolic syndrome.

## Background

It is well established that obesity is an important risk factor for structural alterations in the knee joint. Numerous studies have demonstrated the role obesity plays in the development of osteoarthritis [1–3], cartilage defects [4–7], bone marrow lesions [6–8], pes anserine syndromes [9], and patello-femoral osteoarthritis [10]. These changes are largely attributed to a combination of increased load and altered joint biomechanics [11].

Increased body weight in obesity leads to changes in both joint loading and joint biomechanics during normal day-to-day activities. Obese individuals walk more slowly with shorter and broader strides, have a longer stance duration and a greater toe-out angle when compared with normal weight individuals [12–14]. Obesity also adversely affects the biomechanics of fat surrounding joints. Healthy adipose expansion relies on the presence of a well-coordinated process including the presence of endothelial precursor cells and pre-adipocytes. However, adipose is poorly oxygenated in the obese state [15–17] and hypoxia has been shown to stimulate the propagation of pro-inflammatory adipokynes [18–22], resulting in adipose tissue fibrosis [23].

**Fig. 1:**
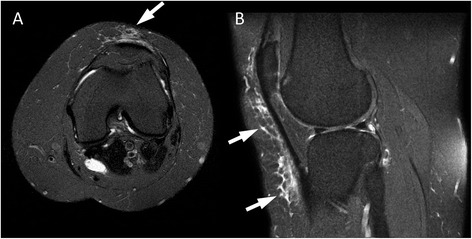
Axial T2-weighted fat-saturated image at the most posterior aspect of the femoral condyles (**a**), and midline sagittal PD-weighted fat-saturated image (**b**) from an MRI examination of the knee of a 46 year old female. Arrows demonstrate abnormal subcutaneous T2 hyper-intense signal anterior to the knee extensor mechanism

An anecdotal observation on magnetic resonance imaging (MRI) is that in some patients with a large amount of subcutaneous fat around the knee, there are sheet-like collections of fluid in the subcutaneous fat particularly over the extensor mechanism (Fig. 1). It may be that the fibrosis of fat associated with chronic obesity predisposes to this. Repeated low level shear forces through relatively stiff fat might then result in chronic interstitial fluid collections.

The primary aim of this study was to test for an association between subcutaneous fluid signal changes and the amount of subcutaneous fat around the knee. The secondary aim was to describe the distribution of these fluid signal changes.

## Methods

Ethical approval was obtained following proportionate review by the NHS National Research Ethics Service (MICRON reference 15/NW/0486). Informed consent was deemed unnecessary for this retrospective case-control study.

### Sample

Mean subcutaneous fat measurements were obtained from a pilot sample of ten patients and used to perform a two tailed sample size calculation for expected mean differences and standard deviations of 0.2, a level of significance of 0.05 and a power of 80%, giving a required sample size of at least 16 patients in each group [24].

A total of 36 MRI examinations were included in the study. Forty-seven examinations exhibiting T2 hyper-intense lesions in the subcutaneous fat around the knee on fat-saturated sequences were identified on our institution’s PACS (examinations between 16 October 2014 and 31 May 2015). Twenty nine were excluded as age and sex-matched controls could not be identified (all female, mean age 53, range 20–66) resulting in eighteen patients included in the study group.

Patients were included if they had undergone an MR examination for chronic knee pain. Patients with a history of acute meniscal, tendon or ligamentous injury or prior knee surgery were excluded, as were those with degenerative or inflammatory arthropathy.

Eighteen age and sex-matched controls without T2 hyper-intense lesions were subsequently identified. The inclusion and exclusion criteria for the control group were the same with the exception that the MR examination had to demonstrate normal fat signal on fat-saturated sequences. The study and control cohorts were selected in a sequential manner until the calculated sample size was exceeded. All cases were independently reviewed by two senior musculoskeletal radiologists (12 and 15 years’ experience) and only included if both agreed that the examinations met the inclusion criteria for each of the cohorts.

### MR imaging acquisition

All patients were imaged on a GE HDX, GE 750w or Siemens Avanto 1.5T MRI machine. The MR acquisitions included sagittal proton density (PD) and intermediate-weighted (IW) fat-saturated sequences, coronal intermediate-weighted and axial T2-weighted fat-saturated sequences. Axial T2-weighted fat-saturated imaging parameters included an echo time (TE) of 85ms, repetition time (TR) of 5186ms, slice thickness of 4.0mm, slice spacing of 0.0mm, an echo train of 11, a field of view 16cm, a matrix size of 320 × 384, and a NEX of 2. Sagittal PD-weighted fat-saturated imaging parameters included a TE of 30ms, TR of 5186ms, slice thickness of 3.5mm, slice spacing of 0.0mm, an echo train of 13, a field of view of 16cm, a matrix size of 320 × 320 and a NEX of 2.

### MR imaging analysis

**Fig. 2:**
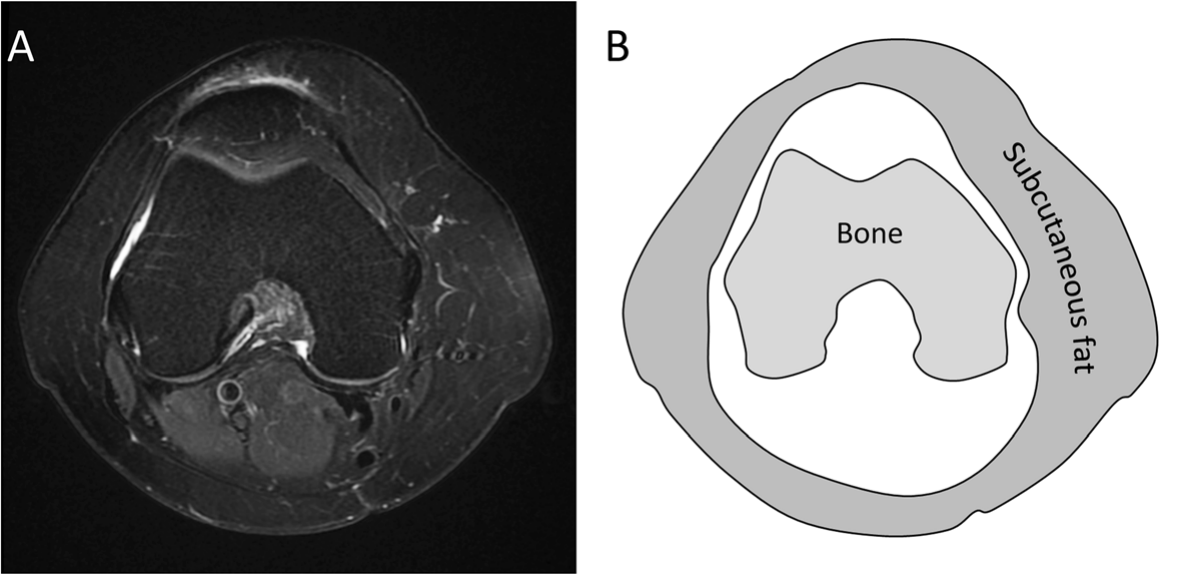
Axial T2-weighted fat-saturated image (**a**) and schematic diagram (**b**) demonstrating regions of interest drawn around bone and subcutaneous fat at the most posterior aspect of the femoral condyles

Cross sectional measurement of bone and subcutaneous fat were performed by two independent observers (radiology trainees) using Osirix MD 7.0 on a diagnostic workstation. A single axial slice for each patient at the most posterior aspect of the femoral condyles on T2-weighted fat-saturated sequences was selected. Regions of interest (ROI) were drawn around bone to calculate the cross-sectional area of the femoral condyles. Two further regions of interest were drawn around the outer circumference of the thigh and the deep fascia. The difference between these two latter ROIs was calculated to be the cross sectional area of subcutaneous fat at this level (Fig. 2). Each measurement was performed twice by each observer with at least two weeks between first and second observations [25].

The location of subcutaneous hyper-intense T2 signal was defined by consensus agreement, measured in a cranio-caudal direction relative to the tibial tuberosity, in the sagittal plane.

### Statistical analysis

All statistical analyses were performed using the statistics programme R [26]. A Shapiro-Wilk test was used to demonstrate normality of data distribution and therefore tests for data comparison. Paired Student t-tests and Wilcoxon Rank Sum tests were used for hypothesis testing. Reliability was measured using intraclass correlation coefficients (ICC) and 95% levels of agreement from Bland-Altman plots. Descriptive statistics were used to define lesion location.

## Results

The study and control groups each comprised 18 females with a mean age of 45, a range of 31–62 years, with 10 left and 8 right knees.

**Fig. 3:**
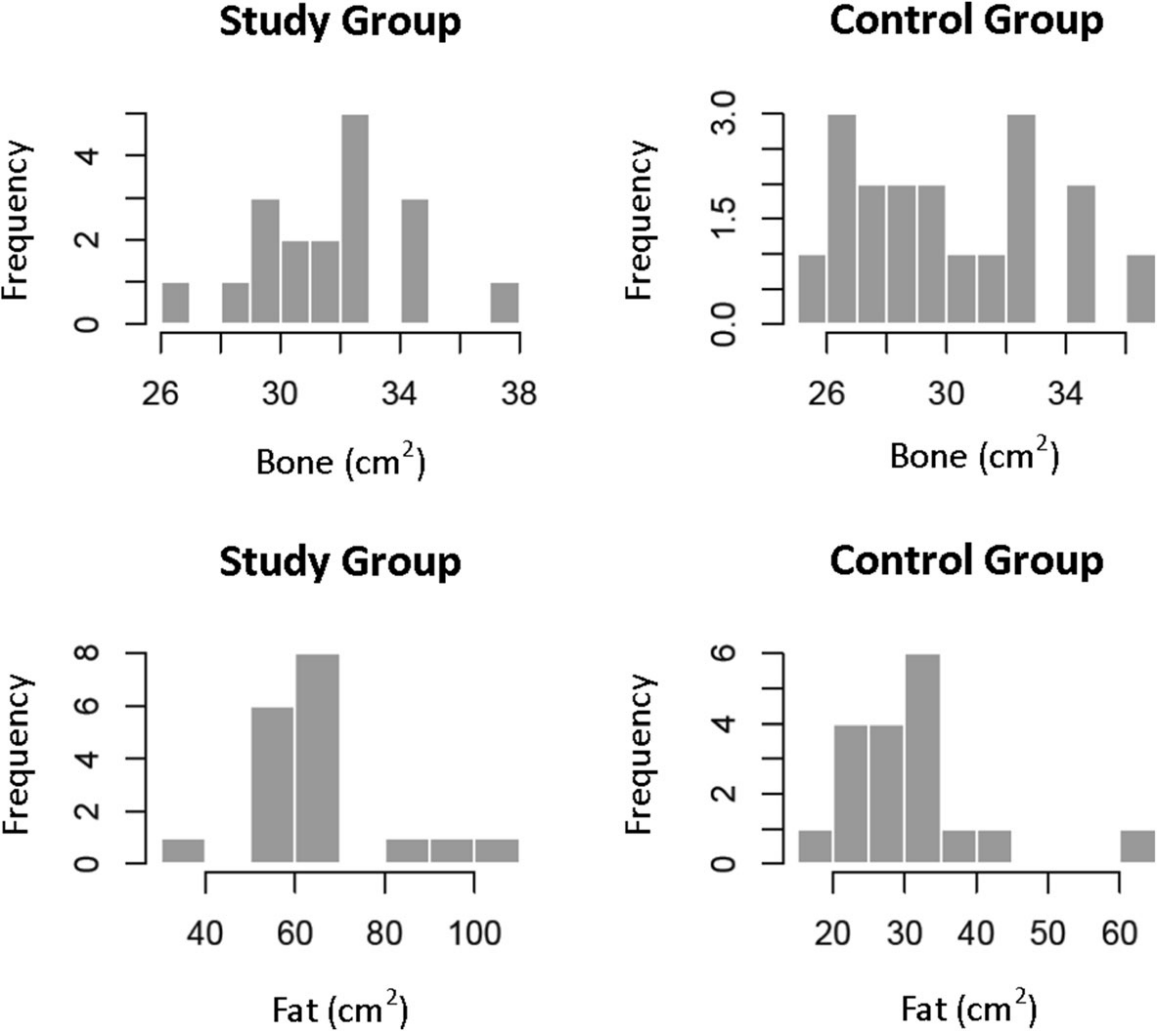
Frequency distribution histograms showing bone and subcutaneous fat measurements in the study and control groups

**Fig. 4:**
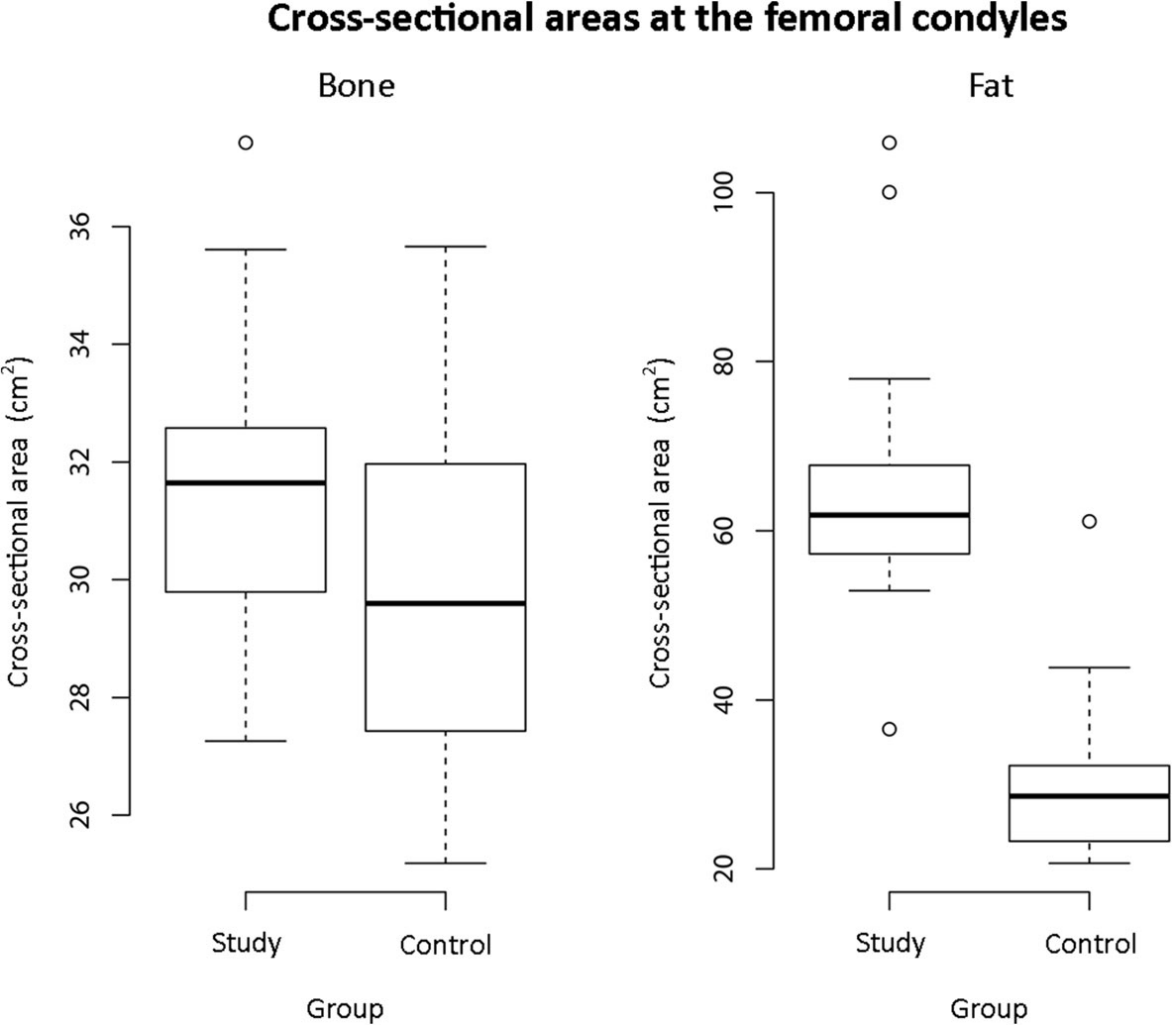
Boxplots demonstrating differences in measured parameters of bone and subcutaneous fat between the study and control groups

The cross-sectional bone measurements conformed to a parametric distribution (*p* = 0.53-0.93) (Fig. 3). The mean area in the study group (first measure performed by observer A) was 31.79cm^2^ (SD 2.57, 95% CI 30.40–32.96 cm^2^) and 30.11cm^2^ (SD 3.20, 95% CI 28.63–31.59cm^2^) in the control group. The difference in means was 1.68cm^2^ and not statistically significant (*p* = 0.09) (Fig. 4). The first results for observer B, and results from the second measurements for both observers were similar (Table 1).

**Table 1:** Cross sectional area of bone (cm^2^)

Review	Mean (cm^2^)	SD	95% CI	DM	95% CI	*p*
A1 Study	31.79	2.57	30.40–32.96	1.68	−0.29–3.65	0.09
A1 Control	30.11	3.20	28.63–31.59			
A2 Study	31.62	2.67	30.38–32.85	1.60	−0.36–3.58	0.10
A2 Control	30.01	3.13	28.56–31.45			
B1 Study	31.75	2.83	30.47–33.05	1.35	−0.44-3.46	0.16
B1 Control	31.40	2.76	29.12–31.67			
B2 Study	31.79	2.79	30.50–33.08	1.51	−0.55-3.24	0.13
B2 Control	30.28	2.96	28.91–31.68			

Cross-sectional subcutaneous fat measurements were not parametric (*p* = 0.0008–0.007) (Fig. 3). The median area in the study group (first measure performed by observer A) was 62.29cm^2^ (IQR 57.09–66.47cm^2^) and 29.39cm^2^ (IQR 24.77–32.33cm^2^) in the control group. The difference in medians was 32.77cm^2^ and statistically significant (*p* < 0.0001) (Fig. 4). The first results for observer B, and results from the second measurements for both observers were similar (Table 2).

**Table 2:** Cross sectional area of subcutaneous fat (cm^2^)

Review	Median (cm^2^)	IQR	*p*
A1 Study	62.29	57.09–66.47	<0.0001
A1 Control	29.39	24.77–32.33	
A2 Study	61.86	57.43–67.54	<0.0001
A2 Control	28.61	23.75–32.00	
B1 Study	63.27	56.16–65.72	<0.0001
B1 Control	30.84	25.23–33.63	
B2 Study	62.45	56.73–65.63	<0.0001
B2 Control	29.91	25.59–33.44	

Inter-rater and intra-rater correlation was “excellent” with coefficients of more than 0.8 for all intraclass correlations [27]. The worst inter-rater reliability was ICC = 0.85 (95% CI 0.64–0.94, *p* < 0.0001) where the mean difference between observers was 0.28cm^2^ (95% limits of agreement -3.50–2.93cm^2^). All other inter-rater ICC scores were better, and can be found in Table 3. The worst intra-rater reliability was ICC = 0.94 (95% CI 0.85–0.98, *p* < 0.0001) with the mean difference between first and second observations of 0.04cm^2^ (95% limits of agreement -1.91–1.83cm^2^). All other intra-rater ICC scores were better, and can be found in Table 4.

**Table 3:** Intra-rater intra-class correlation

Tissue	Review	Difference (cm^2^)	95% LA	ICC	95% CI
Bone	A1/A2 Study	0.17	−1.01–1.35	0.97	0.93–0.99
	A1/A2 Control	0.10	−1.01–1.22	0.98	0.96–0.99
	B1/B2 Study	0.04	−1.91–1.83	0.94	0.85–0.98
	B1/B2 Control	0.11	−1.71–1.94	0.95	0.86–0.98
Fat	A1/A2 Study	0.14	−2.95–3.2	0.99	0.99–1.00
	A1/A2 Control	0.25	−2.37–2.83	0.99	0.97–0.99
	B1/B2 Study	0.33	−3.60–4.26	0.99	0.98–1.00
	B1/B2 Control	0.21	−3.46–3.05	0.99	0.96–0.99

**Table 4:** Inter-rater intra-class correlation

Tissue	Review	Difference (cm^2^)	95% LA	ICC	95% CI
Bone	A1/B1 Study	0.04	−1.48–1.57	0.96	0.89–0.98
	A1/B1 Control	0.28	−3.50–2.93	0.85	0.64–0.94
	A2/B2 Study	0.17	−1.66–1.25	0.96	0.91–0.99
	A2/B2 Control	0.27	−2.26–2.80	0.91	0.78–0.97
Fat	A1/B1 Study	0.24	−2.75–3.23	0.99	0.97–0.99
	A1/B1 Control	1.00	−4.11–2.12	0.99	0.96–0.99
	A2/B2 Study	0.42	−3.45–4.31	0.99	0.98–1.00
	A2/B2 Control	1.46	−5.06–2.15	0.98	0.95–0.99

Key: *A* observer A, *B* observer B, *SD* standard deviation, *DM* difference in means, *CI* confidence interval, *IQR* inter-quartile range, *LA* limits of agreement, *ICC* intra-class correlation coefficient

**Fig. 5:**
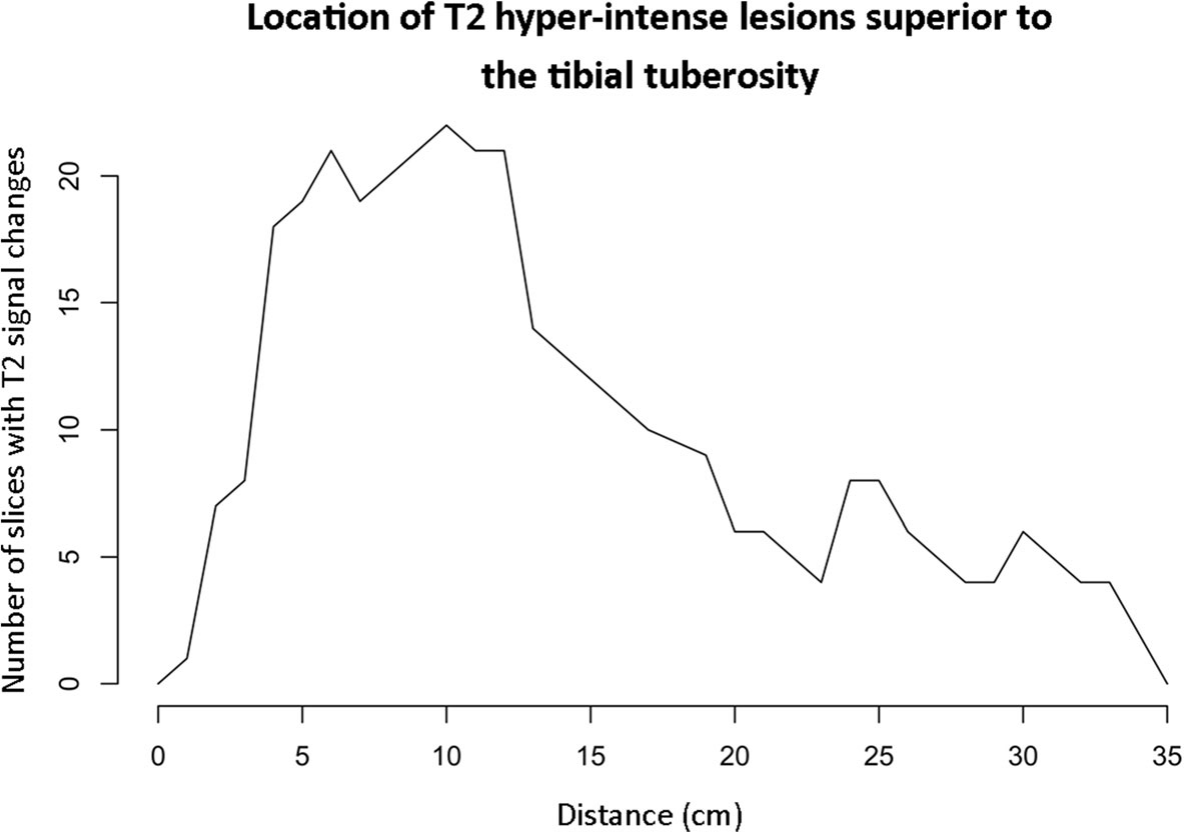
Frequency polygon demonstrating the location of T2 hyper-intense lesions in the study group relative to the tibial tuberosity

There was a non-parametric craniocaudal distribution of T2 hyper-intense signal change, with a median location of 10.4cm (IQR 6.5–16.9cm) superior to the tibial tuberosity (Fig. 5).

## Discussion

This study found that patients demonstrating subcutaneous hyper-intense signal on T2-weighted fat-saturated MRI had significantly more subcutaneous fat around the knee when compared to controls. This suggests that there is an association between the amount of subcutaneous fat and subcutaneous oedema. The cross-sectional area of bone was not statistically different between the study and control groups, suggesting they were comparable in measures other than subcutaneous fat. Most of the fluid signal changes were located 10cm proximal to the tibial tuberosity indicating that this is mainly a pre-patella phenomenon.

All of the cases identified in this retrospective study were women with a mean age in the fifth decade. The female preponderance may be accounted for by the fact that excess adipose tissue tends to be peripherally distributed in females and centrally in males [28]. Even so, measures of appendicular subcutaneous fat are considered to be reasonable surrogate measures of body mass index (BMI) [29, 30]. The age distribution might on first inspection suggest that this is a finding most prevalent in middle age patients. However we were unable to find suitable controls—without any subcutaneous T2 hyper-intense signal changes on their MR examination—for some of the older subjects who then had to be excluded from the study. Therefore both increasing age and subcutaneous fat content may be associated with fluid signal changes in subcutaneous fat.

An association between fluid in the subcutaneous fat and obesity does not necessarily imply causation but there is a mechanism that may explain this association. After eight to 10 years of obesity, individuals develop metabolic syndrome in which the blood supply to adipocytes is reduced causing hypoxia leading to the over-expression of pro-inflammatory cytokines [15–21]. The over-expression of these inflammatory mediators causes macrophage infiltration, extracellular matrix protein up-regulation and fibrosis [23]. A positive feedback loop is accelerated where inflammation in fat propagates to a systemic state of inflammation which can further be attributed to the development of other comorbidities related to obesity [18–20, 31].

Acute injuries of normal subcutaneous fat can result in a Morel-Lavallée lesion, which is a closed de-gloving injury that occurs as a result of blunt trauma. Tangential sheer forces between the subcutaneous tissues and underlying fascia cause a disruption in the vascular and lymphatic supply to the overlying tissues, creating a potential space which fills with blood, lymphatic fluid and necrotic fat [32–34]. Subsequently, blood is largely resorbed and replaced with serosanguinous fluid. Eventually, a capsule forms around the collection meaning the lesion persists [35–37]. The Morel-Lavallée lesion most commonly occurs overlying the greater trochanters, owing to their susceptibility to trauma [27] but has also been reported around the knee [38, 39] and lumbar spine [40].

The development of sheets of fluid in subcutaneous fat of obese patients may then be the result of a chronic shearing injury similar to a Morel-Lavallée lesion [32–34, 36, 38]. In contrast the chronic lesions identified in this study comprised fluid signal but were not well defined and not encapsulated.

If this mechanism of injury is valid then the results of this study would suggest that the shearing forces are greatest over the extensor surface anterior to the patella. To our knowledge there are no published measures of normal soft tissue shearing forces around the knee with which to compare our findings. It is possible that these chronic shearing injuries are in themselves a source of chronic knee pain but this has not been addressed by the current study.

The study is limited by the retrospective design and as a result is prone to selection bias. All subjects had each presented for MRI examination with chronic knee pain, and therefore were a subset of a normal population. The confidence intervals for the means and medians of the study and control groups were narrow suggesting that the sample size was sufficient to provide a good representation of the study population. However this does not mean that these findings can necessarily be generalized to an asymptomatic obese population.

Although the primary endpoint measures were dependent on manually derived ROIs, and the inherent variability associated with these, the measures of inter and intra-rater reproducibility were excellent, and the 95% limits of agreement were narrow, indicating that these are reliable results.

## Conclusion

In conclusion subcutaneous fluid demonstrated on MRI examination of the knee, in patients with chronic knee pain, is associated with increased volumes of subcutaneous fat and is predominantly anterior to the patella, 10cm proximal to the tibial tuberosity.

## Abbreviations

CI: Confidence interval
DM: Difference in means
ICC: Intra-class correlation coefficient
IQR: Interquartile range
IW: Intermediate weighted imaging
LA: Limits of agreement
MRI: Magnetic resonance imaging
NEX: Number of excitations
PD: Proton density weighted imaging
ROI: Region of interest
SD: Standard deviation
TE: Echo Time
TR: Repetition time
