# Erratum to: Fluid signal changes around the knee on MRI are associated with increased volumes of subcutaneous fat: a case-control study

**DOI:** 10.1186/s12891-016-1361-8

**Published:** 2017-01-04

**Authors:** Trevor Gaunt, Frank Carey, John Cahir, Andoni Toms

**Affiliations:** Norwich Radiology Academy, Norfolk and Norwich University Hospitals NHS Foundation Trust, Colney Lane, Norwich, Norfolk NR4 7UB UK

## Erratum

After publication of the original article [1], it became apparent that the family and given names of the corresponding author had been inadvertently switched during production of the manuscript. Trevor Gaunt’s name appears correctly in this erratum, and the author list in the original article has been updated.
